# Novel SARS-like Betacoronaviruses in Bats, China, 2011

**DOI:** 10.3201/eid1906.121648

**Published:** 2013-06

**Authors:** Li Yang, Zhiqiang Wu, Xianwen Ren, Fan Yang, Guimei He, Junpeng Zhang, Jie Dong, Lilian Sun, Yafang Zhu, Jiang Du, Shuyi Zhang, Qi Jin

**Affiliations:** Ministry of Health Key Laboratory of Systems Biology of Pathogens, Beijing, China (L. Yang, Z. Wu, X. Ren, F. Yang, J. Dong, L. Sun, Y. Zhu, J. Du, Q. Jin);; Institute of Pathogen Biology, Beijing (L. Yang, Z. Wu, X. Ren, F. Yang, J. Dong, L. Sun, Y. Zhu, J. Du, Q. Jin);; East China Normal University, Shanghai, China (G. He, J. Zhang, S. Zhang)

**Keywords:** Coronavirus, Chiroptera, SARS virus, China, viruses, bats

## Abstract

To clarify the evolutionary relationships among betavoronaviruses that infect bats, we analyzed samples collected during 2010–2011 from 14 insectivorous bat species in China. We identified complete genomes of 2 novel betacoronaviruses in *Rhinolophus pusillus* and *Chaerephon plicata* bats, which showed close genetic relationships with severe acute respiratory syndrome coronaviruses.

The 2003 outbreak of severe acute respiratory syndrome (SARS) was caused by a novel betacoronavirus and rapidly spread globally, causing ≈8,000 cases and nearly 900 deaths (*1*,*2*). In June 2012, a novel betacoronavirus (called human coronavirus EMC [HCoV-EMC]) also was isolated from the sputum of a patient from Saudi Arabia who died of pneumonia and renal failure (*3*). Similar viruses were detected in 2 additional patients who had severe pneumonia in Qatar in September 2012 and in Saudi Arabia in November 2012 (*4*,*5*). The clinical picture was remarkably similar to that of SARS and illustrates the epidemic potential of a novel coronavirus (CoV) to threaten global health. SARS-CoVs and HCoV-EMC were suspected of spreading from bats to humans because these CoVs were most closely related to bat CoVs (*1*,*4*). To clarify the evolutionary relationships among betavoronaviruses that infect bats, we analyzed samples collected during 2010–2011 from 14 insectivorous bat species common in 8 provinces in China.

## The Study

We obtained pharyngeal and anal swab specimens of 414 insectivorous bats. Samples of each species were pooled and then processed with a viral particle–protected nucleic acid purification method (*6*). The extracted RNA and DNA were amplified by sequence-independent PCR. The amplified viral nucleic acid libraries of the bat species were then sequenced with the Illumina/Solexa GAII sequencer (Illumina, San Diego, CA, USA). Those reads generated by the Illumina/Solexa GAII with length of 80 bases were directly aligned to the protein sequences in the National Center for Biotechnology Information nonredundant protein database by the blastx program in the BLAST software package, version 2.2.22 (www.ncbi.nlm.nih.gov/blast) with parameters “-e 1e-5 -F T -b 10 -v 10.” No assembly was performed before alignment. Sequence similarity–based taxonomic assignments were conducted as described (*7*). We found 1,075 reads of betacoronavirus in *Rhinolophus pusillus* bats in Shaanxi and 92 reads of betacoronavirus in *Chaerephon plicata* bats in Yunnan.

We estimated the approximate locations of those reads on the CoV genome and their relative distances on the basis of alignment results exported with MEGAN 4–MetaGenome Analyzer (http://ab.inf.uni-tuebingen.de/software/megan/). The located reads were then used for reads-based nested PCR to identify genomic sequences. We established the complete genome sequences of 2 betacoronaviruses (Bat Rp-coronavirus/Shaanxi2011 and Bat Cp-coronavirus/Yunnan2011), which are 29,484 nt and 29,452 nt, respectively. The G+C content of Bat Rp-coronavirus/Shaanxi2011 and Bat Cp-coronavirus/Yunnan2011 is 41.6% and 40.9%, respectively.

We conducted complete genome comparison and phylogenetic analysis on the basis of polymerase and spike protein. Pairwise genome sequence alignment was conducted by using EMBOSS Needle software (www.ebi.ac.uk/Tools/psa/emboss_needle/) with default parameters. The overall nucleotide sequences between Bat Rp-coronavirus/Shaanxi2011 and Bat Cp-coronavirus/Yunnan2011 indicated 88.7% nt identity. They shared 87.4%–89.5% nt identity with SARS-CoV, 88%–89.9% nt identity with the bat SARS-like CoV (bat SARS-CoV Rm1), and 87.6%–89.6% nt identity with the civet SARS-like CoV (civet SARS-CoV SZ16). On the other hand, comparison between the betacoronavirus genomes and human betacoronavirus (HCoV-OC43) showed only 49.9%–50.4% nt overall identity, whereas the betacoronavirus genomes and HCoV-EMC showed 52.1% nt overall identity.

**Figure 1:**
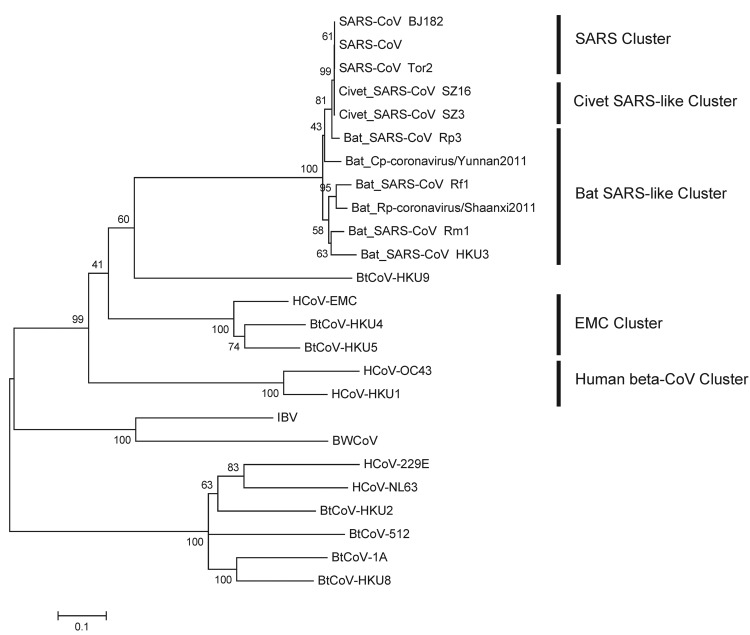
Phylogenetic tree of novel betacoronaviruses based on the nucleotide sequence of the *RdRp* gene. The following coronaviruses (CoVs) and GenBank accession numbers were used: bat severe acute respiratory syndrome CoV Rm1 (bat SARS-CoV Rm1; DQ412043), bat SARS-CoV Rp3 (DQ071615), bat SARS-CoV Rf1 (DQ412042), bat SARS-CoV HKU3 (DQ022305),SARS-CoV isolate Tor2/FP1–10895 (SARS-CoV Tor2; JX163925), SARS-CoV BJ182–12 (SARS-CoV BJ182; EU371564), SARS-CoV (NC004718), civet SARS-CoV SZ3 (AY304486), civet SARS-CoV SZ16 (AY304488), bat CoV HKU9 (BtCoV-HKU9; EF065513), bat CoV HKU4 (BtCoV-HKU4; EF065505), bat CoV HKU5 (BtCoV-HKU5; EF065509), human betacoronvirus 2c EMC/2012 (HCoV-EMC; JX869059), human CoV OC43 (HCoV-OC43; NC005147), HCoV-HKU1 (NC006577), bat coronavirus HKU2 (BtCoV-HKU2; NC009988), bat coronavirus 1A (BtCoV-1A; NC010437), HCoV-229E (NC002645), HCoV-NL63 (NC005831), bat CoV HKU8 (BtCoV-HKU8; NC010438), scotophilus bat CoV 512 (BtCoV-512; NC009657), avian infectious bronchitis virus (IBV; NC001451), beluga whale CoV SW1 (BWCoV; NC010646). Scale bar indicates genetic distance estimated by using TN93+G+I model implemented in MEGA5 (www.megasoftware.net).

The RNA-dependent RNA polymerase (RdRp, the 12th nonstructural protein codified to open reading frame 1a,b) is a highly conserved gene of CoVs, which is frequently used for phylogenetic comparison (*8*,*9*). MEGA5.0 (www.megasoftware.net) was used to construct the phylogenetic trees on the basis of the nucleotide sequences and deduced amino acid sequences. First, we used the MUSCLE package and default parameters (www.megasoftware.net/) to construct the alignment. The best substitution model was then evaluated with the Model Selection package implemented in MEGA5. Finally, we used the maximum-likelihood method with an appropriate model to process the phylogenetic analysis with 1,000 bootstrap replicates. We constructed a phylogenetic tree based on the nucleotide sequences of the *RdRp* gene to show the evolutionary relationship between these 2 betacoronaviruses and other CoVs (Figure 1). Reference CoV genome sequences were downloaded from GenBank and aligned with the fragments of the newly discovered CoVs. The *RdRp* genes of Bat Rp-coronavirus/Shaanxi2011 and Bat Cp-coronavirus/Yunnan2011 were highly similar, sharing 93.1% nt identity. The phylogenetic analysis demonstrated that betacoronaviruses and the bat SARS-like CoVs in our study are clustered (93.1%–93.4% nt identity) and are close in distance to SARS-CoVs (92.9%–94.8% nt identity) and civet SARS-like CoVs (93.1%–94.8% nt identity) but that bat CoV (BtCoV-HKU9) and HCoV-OC43 are placed among the relatively distant groups (65.8%–65.9% and 62.9%–63.5% nt identities with the betacoronaviruses, respectively). Therefore, collectively we called these betacoronaviruses and bat SARS-like CoVs the bat SARS-like cluster of CoVs. Bat Rp-coronavirus/Shaanxi2011 and Bat Cp-coronavirus/Yunnan2011 showed little genetic similarity (<66.2%–67.3% nt identity) to HCoV-EMC.

**Figure 2:**
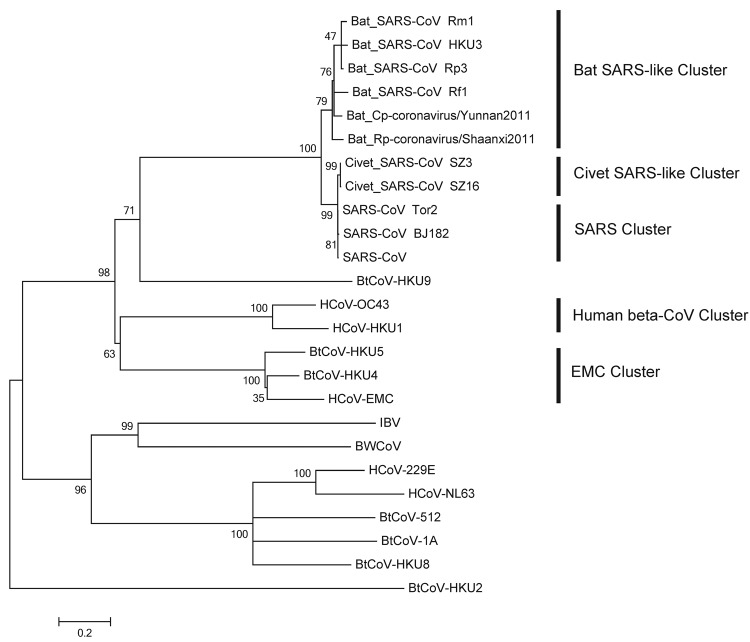
Phylogenetic tree of novel betacoronaviruses based on the deduced amino acid sequence of spike protein. SARS, severe acute respiratory syndrome; CoV, coronavirus; HCoV, human CoV; BtCoV, bat CoV; BWCoV, beluga whale CoV; IBV, avian infectious bronchitis. Scale bar indicates genetic distance estimated by using WAG+G+I+F model implemented in MEGA5 (www.megasoftware.net).

The spike proteins of CoVs are responsible for receptor binding and host species adaptation, and their genes therefore constitute one of the most variable regions within CoV genomes (*10*,*11*). The phylogenetic tree based on the amino acid sequences of spike protein (Figure 2) suggests that the selected betacoronaviruses were mainly divided into 5 clusters: SARS cluster; bat SARS-like cluster; civet SARS-like cluster; human betacoronavirus cluster; and EMC cluster. Bat Rp-coronavirus/Shaanxi2011 and Bat Cp-coronavirus/Yunnan2011 shared 89.4% aa identity in spike proteins, which consisted of 1,240 aa and 1,241 aa, respectively. The spike proteins of the CoVs in our analysis have 89.8%–92.7% aa identity with those of bat SARS-like CoVs, with substantial similarity in the receptor-binding domain. The close relationship also was observed with the SARS-CoVs (79.2%–79.4% aa identity) and civet SARS-like CoVs (78.9%–79.1% aa identity). In contrast, the human betacoronaviruses and EMC cluster formed separate clusters distinct from SARS-related CoVs that showed only 27.8%–29.4% aa and 28.8%–30.5% aa identities with the betacoronaviruses, respectively, in our analysis. The genome sequences reported here have been deposited into GenBank (accession nos. JX993987–JX993988).

## Conclusions

The recent fatal human infection caused by HCoV-EMC has boosted interest in the discovery of novel CoVs in humans and animals. HCoV-EMC is a novel betacoronavirus, and its closest known relatives are BtCoVs HKU4, and HKU5, which have been detected in Hong Kong only in bats (*12*), the same animal from which SARS is believed to have originated. Bats are increasingly recognized as natural reservoirs of CoVs and may serve as intermediate hosts for interspecies transmission of SARS-CoVs (*10*,*13*). Different bat populations from various countries harbor diverse CoVs that have a high frequency of recombination and mutation rates that enable them to adapt to new hosts and ecologic niches (*14*,*15*). Therefore, continuous studies of CoVs from different bat species and different countries would help better prevent the new global pandemics resulting from novel viral infection.

We detected and characterized 2 novel betacoronaviruses—Bat Rp-coronavirus/Shaanxi2011 in *R. pusillus* bats and Bat Cp-coronavirus/Yunnan2011 in *C. plicata* bats—in China. The high similarity shown by phylogenetic analysis confirmed the close genetic relationship among the CoVs (SARS-like CoVs and SARS-CoVs) that we analyzed. In contrast, Bat Rp-coronavirus/Shaanxi2011 and Bat Cp-coronavirus/Yunnan2011 showed little genetic similarity with human betacoronaviruses and HCoV-EMC. Although several CoVs are found in horseshoe bats (*Rhinolophus* spp.), to our knowledge, the SARS-like CoVs in *R. pusillus* and *C. plicata* bats in China have not been identified. The description presented here will further the understanding of CoVs distribution in different bat species found in human habitats and provide clues for rapid response to potential public health threats.

## References

[1] Peiris JS, Yuen KY, Osterhaus AD, Stohr K. The severe acute respiratory syndrome. N Engl J Med. 2003;349:2431–41. 10.1056/NEJMra032498 14681510

[2] Drosten C, Gunther S, Preiser W, van der Werf S, Brodt HR, Becker S, Identification of a novel coronavirus in patients with severe acute respiratory syndrome. N Engl J Med. 2003;348:1967–76. 10.1056/NEJMoa030747 12690091

[3] Zaki AM, van Boheemen S, Bestebroer TM, Osterhaus AD, Fouchier RA. Isolation of a novel coronavirus from a man with pneumonia in Saudi Arabia. N Engl J Med. 2012;367:1814–20. 10.1056/NEJMoa1211721 23075143

[4] Corman V, Eckerle I, Bleicker T, Zaki A, Landt O, Eschbach-Bludau M, Detection of a novel human coronavirus by real-time reverse-transcription polymerase chain reaction. Euro Surveill. 2012;17:20285 . 23041020 10.2807/ese.17.39.20285-en

[5] ProMEDmail. Novel coronavirus—Saudi Arabia: new case. ProMED-mail 2012 Nov 04 [cited 2012 Nov 4]. http://www.promedmail.org/, article no. 20121104.1391285.

[6] Wu Z, Ren X, Yang L, Hu Y, Yang J, He G, Virome analysis for identification of novel mammalian viruses in bat species from Chinese provinces. J Virol. 2012;86:10999–1012. 10.1128/JVI.01394-12 22855479 PMC3457178

[7] Yang J, Yang F, Ren L, Xiong Z, Wu Z, Dong J, Unbiased parallel detection of viral pathogens in clinical samples by use of a metagenomic approach. J Clin Microbiol. 2011;49:3463–9. 10.1128/JCM.00273-11 21813714 PMC3187305

[8] Woo PC, Lau SK, Chu CM, Chan KH, Tsoi HW, Huang Y, Characterization and complete genome sequence of a novel coronavirus, coronavirus HKU1, from patients with pneumonia. J Virol. 2005;79:884–95. 10.1128/JVI.79.2.884-895.2005 15613317 PMC538593

[9] Lau SK, Li KS, Tsang AK, Shek CT, Wang M, Choi GK, Recent transmission of a novel alphacoronavirus, bat coronavirus HKU10, from Leschenault’s rousettes to Pomona leaf-nosed bats: first evidence of interspecies transmission of coronavirus between bats of different suborders. J Virol. 2012;86:11906–18. 10.1128/JVI.01305-12 22933277 PMC3486284

[10] Li W, Shi Z, Yu M, Ren W, Smith C, Epstein JH, Bats are natural reservoirs of SARS-like coronaviruses. Science. 2005;310:676–9. 10.1126/science.1118391 16195424

[11] Lau SK, Woo PC, Li KS, Huang Y, Wang M, Lam CS, Complete genome sequence of bat coronavirus HKU2 from Chinese horseshoe bats revealed a much smaller spike gene with a different evolutionary lineage from the rest of the genome. Virology. 2007;367:428–39. 10.1016/j.virol.2007.06.009 17617433 PMC7103351

[12] Woo PC, Lau SK, Li KS, Poon RW, Wong BH, Tsoi HW, Molecular diversity of coronaviruses in bats. Virology. 2006;351:180–7. 10.1016/j.virol.2006.02.041 16647731 PMC7111821

[13] Balboni A, Battilani M, Prosperi S. The SARS-like coronaviruses: the role of bats and evolutionary relationships with SARS coronavirus. New Microbiol. 2012;35:1–16 . 22378548

[14] Lau SK, Lee P, Tsang AK, Yip CC, Tse H, Lee RA, Molecular epidemiology of human coronavirus OC43 reveals evolution of different genotypes over time and recent emergence of a novel genotype due to natural recombination. J Virol. 2011;85:11325–37. 10.1128/JVI.05512-11 21849456 PMC3194943

[15] Woo PC, Lau SK, Huang Y, Yuen KY. Coronavirus diversity, phylogeny and interspecies jumping. Exp Biol Med (Maywood). 2009;234:1117–27. 10.3181/0903-MR-94 19546349

